# High Strength and Fracture Resistance of Reduced-Activity W-Ta-Ti-V-Zr High-Entropy Alloy for Fusion Energy Applications

**DOI:** 10.3390/e27080777

**Published:** 2025-07-23

**Authors:** Siva Shankar Alla, Blake Kourosh Emad, Sundeep Mukherjee

**Affiliations:** Department of Materials Science and Engineering, University of North Texas, Denton, TX 76203, USA; sivashankaralla@my.unt.edu (S.S.A.); blakeemad@my.unt.edu (B.K.E.)

**Keywords:** plasma-facing component, high-entropy alloy, refractory, reduced activity, high toughness

## Abstract

Refractory high-entropy alloys (HEAs) are promising candidates for next-generation nuclear applications, particularly fusion reactors, due to their excellent high-temperature mechanical properties and irradiation resistance. Here, the microstructure and mechanical behavior were investigated for an equimolar WTaTiVZr HEA, designed from a palette of low-activation elements. The as-cast alloy exhibited a dendritic microstructure composed of W-Ta rich dendrites and Zr-Ti-V rich inter-dendritic regions, both possessing a body-centered cubic (BCC) crystal structure. Room temperature bulk compression tests showed ultra-high strength of around 1.6 GPa and plastic strain ~6%, with fracture surfaces showing cleavage facets. The alloy also demonstrated excellent high-temperature strength of ~650 MPa at 500 °C. Scratch-based fracture toughness was ~38 MPa√m for the as-cast WTaTiVZr HEA compared to ~25 MPa√m for commercially used pure tungsten. This higher value of fracture toughness indicates superior damage tolerance relative to commercially used pure tungsten. These results highlight the alloy’s potential as a low-activation structural material for high-temperature plasma-facing components (PFCs) in fusion reactors.

## 1. Introduction

The operating environment in a fusion reactor places extreme demands on the materials used, particularly plasma-facing components (PFCs), such as the first wall and the divertor. These components are exposed to a combination of high heat flux, particle bombardment, and neutron irradiation. Among the PFCs, the divertor plays a key role in handling power and particle exhaust, removing helium ash, and limiting impurity transport into the plasma core. This function concentrates the most intense plasma–material interactions into a confined region that must be carefully designed to maintain performance under sustained exposure [1,2]. Tungsten (W) has emerged as a leading choice for plasma-facing applications, particularly in the first wall and divertor, because of its high melting point, high thermal conductivity, and low sputtering yield. However, pure W lacks long-term irradiation resistance, is susceptible to oxidation, and has poor toughness [3,4,5]. Tungsten-based high-entropy alloys (HEAs) composed of other refractory elements along with tungsten have gained significant attention in recent years for fusion energy applications because of their excellent high-temperature strength, thermal stability, and irradiation resistance [6,7,8,9]. Previous studies on W-based HEAs have demonstrated several promising characteristics, including microstructural stability, high-temperature strength, and “cocktail effects” arising from the multi-principal element design [6,7,10]. Additionally, high lattice distortion and sluggish diffusion in these alloy systems may contribute to improved irradiation tolerance. To evaluate their suitability for structural applications in fusion environments, it is important to understand how microstructural features influence their high-temperature mechanical behavior. However, most of the W-based HEAs developed so far have niobium (Nb), molybdenum (Mo), chromium (Cr), and nickel (Ni) as constituents. While elements, such as Nb, Mo, Cr, and Ni, have high melting temperatures, their suitability for PFCs, particularly the first wall and blanket, is limited. This is primarily due to their neutron activation behavior, which results in the formation of long-lived radioactive waste, thereby complicating disposal. Additionally, Nb is prone to hydrogen embrittlement, Cr exhibits poor resistance to liquid metal corrosion, and Mo suffers from irradiation-induced embrittlement [11,12,13]. Structural materials used in PFCs must not only possess superior thermal and mechanical properties but also exhibit low activation and rapid decay of induced radioactivity to achieve safe “hands-on” handling and recycling [14]. This requires careful selection of alloying elements for W-based HEAs to minimize the formation of long-lived radioisotopes.

Here, the microstructure and high-temperature mechanical properties of an equimolar WTaTiVZr HEA with low-activity constituent elements were investigated and compared to commercially used pure tungsten. All the constituent elements selected in our WTaTiVZr HEA possess melting points above 1650 °C and show relatively low neutron activation. Importantly, they decay to safe “hands-on” dose levels significantly faster than other commonly used refractory elements such as Mo, Nb, and Hf [10,15]. These attributes, including thermal stability and reduced activation, are particularly advantageous for enhancing the safety and recyclability of structural materials in plasma-facing components. We have previously reported on the excellent creep resistance and molten salt corrosion resistance of this alloy system [15,16]. However, there are no reports and limited understanding of their plastic strain and toughness, which are critical design criteria for structural materials used in PFCs. To address these knowledge gaps, we have used a combination of bulk compression at room temperature, plastometer-based indentation to determine strength and plastic strain up to 500 °C, and scratch-based fracture toughness measurements for our WTaTiVZr HEA. Standard methods for measuring fracture toughness (ASTM-E399) [17] use specific test geometries and assume plane strain conditions, which are geometry dependent [18,19]. Estimating the fracture toughness of tungsten and refractory high-entropy alloys using compact tension (CT) [20] or single-edge notch bending (SENB) [21] is challenging, as they tend to fracture at room temperature without noticeable plastic deformation, making crack initiation and propagation difficult to control during testing. The scratch-based measurement approach offers several advantages over conventional fracture toughness tests, particularly for brittle and hard materials such as tungsten and refractory high-entropy alloys (RHEAs). It generates a controlled and highly localized stress field, enabling stable crack initiation and propagation without sudden failure [22,23]. While standard methods for measuring fracture toughness rely on stable plastic deformation at the crack tip and assume elastic–plastic behavior, these conditions are often not met in tungsten, where crack initiation can be abrupt and difficult to control. Moreover, the high hardness and density of such materials make it challenging to fabricate precise and flaw-free notched specimens, often compromising test validity. In contrast, the scratch method requires minimal sample preparation, is effective on small volumes, and is less sensitive to geometric constraints or surface flaws. Fracture toughness is estimated from the energy required to initiate and propagate cracks, which makes it especially effective for brittle materials. Furthermore, the technique is minimally destructive and surface-sensitive, making it ideal for high-throughput evaluation of fracture resistance in structural materials [22,23].

## 2. Materials and Methods

The WTaTiVZr alloy with an equimolar proportion of constituent elements was synthesized via arc melting using elemental powders of >99.99% purity. The resulting ingot was re-melted at least five times to ensure chemical homogeneity. For comparison, a pure tungsten plate was obtained from AEM Metal (Changsha, China) that was fabricated through sintering of pure elemental powder. Phase analysis was performed using a Bruker D6 Phaser X-ray diffractometer (Bruker, Minneapolis, MN, USA) with a Cu Kα source operating at 40 kV and 44 mA. Scans were performed over a 2θ range of 20–90° at a step rate of 1° per minute. Microstructural characterization was performed using a Thermo Scientific Quattro environmental scanning electron microscope (Thermo Fisher Scientific, Waltham, MA, USA) equipped with an energy-dispersive X-ray spectroscopy (EDS) system for elemental analysis. Uniaxial compression tests were performed on cylindrical specimens 4 mm in diameter and 4 mm in height using an MTS 322 servo-hydraulic testing system (MTS, Eden Prairie, Minnesota, MN, USA). The tests were carried out at a constant engineering strain rate of 0.001 s^−1^ under quasi-static conditions. At least three samples were tested to ensure the reproducibility of mechanical behavior.

High-temperature mechanical behavior was assessed through plastometer-based indentation testing on cube specimens measuring 5 mm × 5 mm × 5 mm over the temperature range of room temperature to 500 °C in 100 °C increments using a Plastometer (PLX-Hot Stage, Cambridge, UK). Further details of the plastometer-based testing procedure are discussed elsewhere [24,25]. A silicon nitride (Si_3_N_4_) indenter with a tip radius of 1 mm was used. The penetration depth-to-radius ratio (δ/R) ranged from 10% to 20%, under applied loads up to 5 kN. Indentation profiles were recorded using a stylus profilometer with a resolution of 1 µm and indent diameters ~1 mm. Multiple indents were made on each sample, and representative profiles were selected to extract the corresponding stress–strain response.

Scratch-based fracture toughness tests were performed using an RTEC Universal Tribometer (RTEC Instruments, San Jose, CA, USA) equipped with a Rockwell C-type Sphero conical diamond stylus (200 μm tip radius, 60° cone angle). A constant normal load of 100 N was applied, selected through preliminary depth calibration tests to produce consistent fracture features across all sample types. Scratching was carried out at a fixed speed of 0.01 mm/s over a 3 mm length. Each test was repeated three times to ensure reproducibility and statistical confidence. The transverse force (*F_T_*) was measured along with the applied normal force (*F_N_*) using a biaxial load cell. Post-scratch characterization involved confocal laser scanning microscopy to capture three-dimensional profiles of the scratch tracks, including the groove perimeter (*P*) and the projected contact area (*A_C_*). These values were used to estimate fracture toughness (*K_C_*) following the method developed by Akono et al. [22]:

(1) $$K_{C}=\frac{F_{T}}{{\left[2PA_{C}\right]}^{\frac{1}{2}}}$$

## 3. Results and Discussion

**Figure 1:**
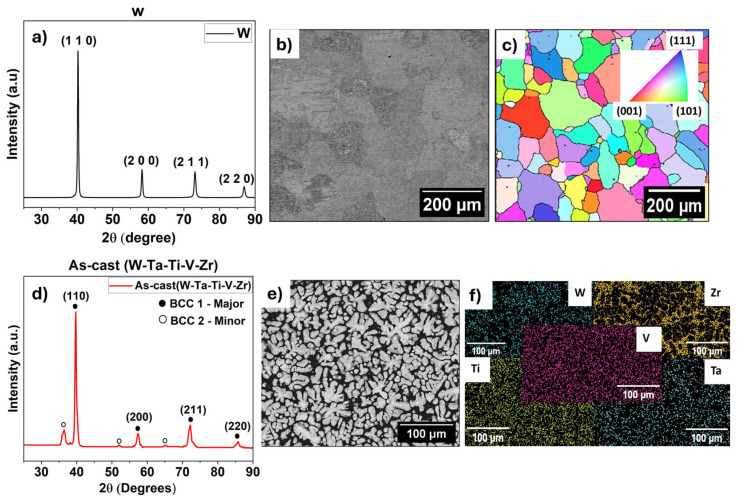
(**a**) XRD pattern of pure tungsten; (**b**,**c**) BSE image showing coarse grain microstructure and EBSD inverse pole figure map of pure tungsten; (**d**) XRD pattern of WTaTiVZr HEA confirming BCC1 and BCC2 phases; (**e**) BSE image of WTaTiVZr HEA showing dendritic microstructure; (**f**) EDS maps showing W-Ta-rich dendrites and Zr-Ti-rich inter-dendritic regions.

Figure 1a–f summarize the microstructural results of commercially used sintered pure tungsten and the as-cast WTaTiVZr HEA. Figure 1a shows the XRD pattern of pure tungsten, indicating a single-phase body-centered cubic (BCC) crystal structure. Figure 1b,c show the SEM and electron backscatter diffraction (EBSD) images of the microstructure, respectively, indicating coarse, equiaxed grains and random orientations. The average grain size for pure tungsten was ~100 ± 20 µm. Figure 1d shows the XRD pattern of our as-cast RHEA. The diffraction pattern shows two sets of BCC peaks, labeled BCC1 (major) and BCC2 (minor), suggesting the presence of BCC phases with slightly different lattice parameters. These phases correspond to the W–Ta-rich dendritic regions and the Zr–Ti-rich inter-dendritic regions. The formation of multiple BCC phases is attributed to elemental segregation during solidification. Figure 1e shows the microstructure of the alloy recorded in backscattered electron (BSE) mode, clearly indicating two distinct phases: a lighter contrast dendritic phase and a darker contrast inter-dendritic phase. Figure 1f shows the EDS maps revealing clear segregation among the constituent elements: W and Ta, being higher melting point elements, are segregated in the dendrites, while Zr and Ti preferentially segregate to the inter-dendritic regions. Vanadium appears evenly distributed between the two BCC phases.

**Figure 2:**
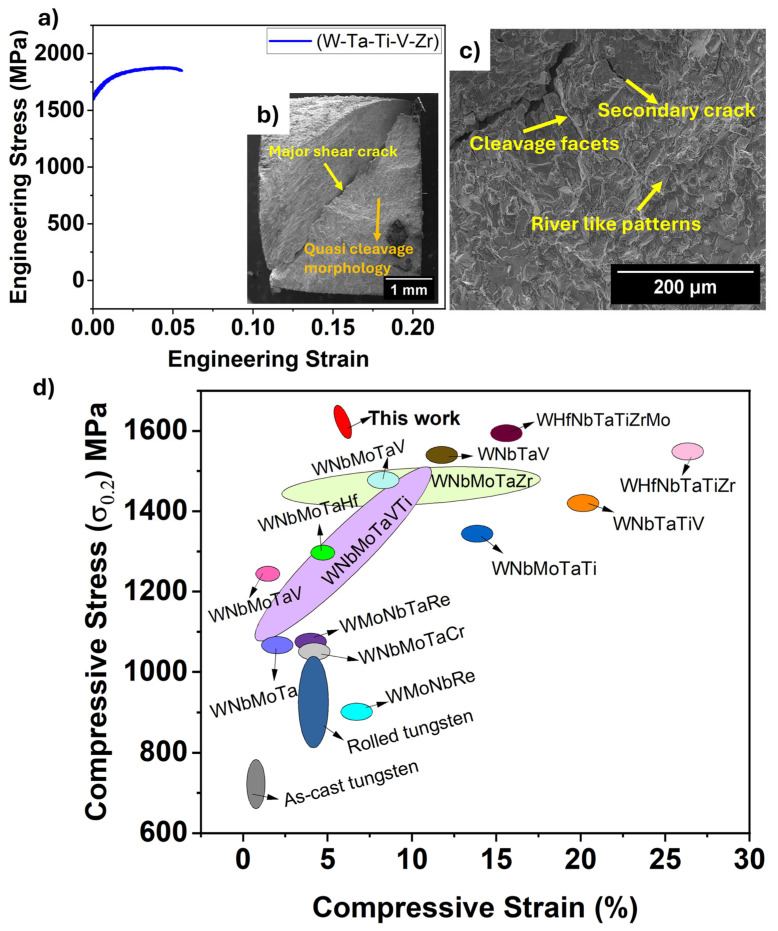
(**a**) Compressive engineering stress–strain curve showing ultra-high YS and limited plasticity of WTaTiVZr HEA. (**b**) Fracture surface with shear crack and quasi-cleavage characteristics. (**c**) Zoomed-in view showing cleavage facets and river patterns indicating brittle failure for our WTaTiVZr HEA. (**d**) Ashby plot showing compressive yield stress and compressive strain of W-based as-cast RHEAs and as-cast tungsten [8,9] compared to our WTaTiVZr HEA.

Figure 2a shows the room temperature compressive engineering stress–strain curve for the WTaTiVZr HEA. It shows a yield strength (YS) of approximately 1600 MPa and an ultimate compressive strength of 1800 MPa. This YS is significantly higher than that of conventional refractory metals, which typically show a YS of 500–800 MPa at room temperature and exceed the strength of several reported refractory HEAs [8,9]. For example, the equiatomic MoNbTaW HEA has a YS of ~996 MPa while TiNbWTaMo reaches ~1455 MPa [26]. The ultra-high strength in the WTaTiVZr alloy may be attributed to solid solution strengthening and lattice distortion effect from the constituent refractory principal elements [9,27,28]. The WTaTiVZr alloy shows a plastic strain of ~6%, a behavior typical of many refractory HEAs, especially those containing W and Mo [9,27,29]. Figure 2b,c show the fracture surface morphology of the alloy, indicating a major shear crack and brittle failure. A zoomed-in view of the fracture surface (Figure 2c) shows quasi-cleavage characteristics, including cleavage facets, river-like pattern, and secondary cracks. This fracture behavior suggests crack propagation along specific crystallographic planes, consistent with trans-granular cleavage observed in BCC HEAs such as MoNbWTa [26]. Ti and V are known to enhance ductility in refractory HEAs; for instance, Ti addition in MoNbWTa HEA has been shown to improve ductility by ~11.5%. However, in the present case, the high fraction of W and Ta appears to suppress such effects, leading to the observed brittle behavior at room temperature. Figure 2d shows an Ashby plot comparing the room temperature compressive yield strength and compressive strain for as-cast tungsten and tungsten-based as-cast RHEAs [8,9]. Although some alloys exhibit higher compressive strains (~25%), they contain elements such as Mo, Nb, and Hf. As discussed earlier, these elements have significant drawbacks for nuclear applications, including long-lived radioactive waste generation and susceptibility to embrittlement and corrosion. In contrast, the WTaTiVZr RHEA shows the highest compressive strength among the reported tungsten-containing as-cast RHEAs and favorable radioactive decay characteristics, making it particularly promising for PFC applications.

**Figure 3:**
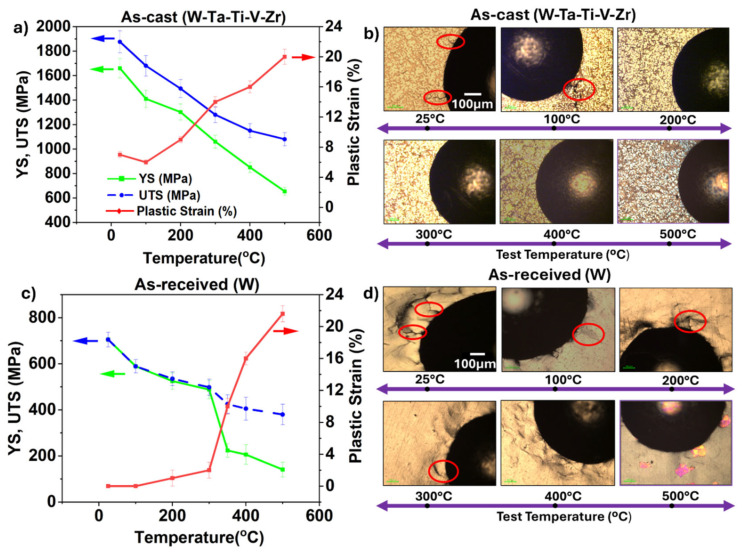
Yield strength, ultimate tensile strength, and plastic strain versus temperature obtained from plastometer indentation for (**a**) WTaTiVZr HEA and (**c**) commercially used pure tungsten. Optical images as a function of temperature of the area around the indents for (**b**) WTaTiVZr HEA and (**d**) commercially used pure tungsten.

Figure 3 shows the variation in yield strength (YS), ultimate tensile strength (UTS), and plastic strain as a function of temperature for the WTaTiVZr HEA versus commercially used pure tungsten measured using plastometer-based indentation. Pure tungsten (Figure 3c) exhibited a YS of approximately 700 MPa with negligible plastic strain. This negligible plasticity may be attributed to the nature of dislocation motion in tungsten. The core of the 1/2⟨111⟩ screw dislocation is spread across multiple {110} and {112} planes, which significantly restricts its mobility and results in high Peierls stress. As the temperature increases, thermal activation reduces the effective Peierls barrier, enabling limited dislocation glide and allowing a gradual increase in plastic strain [30,31,32,33]. However, plastic strain in pure tungsten remains negligible up to 300 °C and increases sharply at ~400 °C. This limited plasticity of tungsten over a wide temperature range is a major concern for fusion systems, as it increases the risk of failure during thermal cycling [14,34]. The WTaTiVZr high-entropy alloy (Figure 3a) showed significantly higher strength compared to pure tungsten over the same temperature range. At room temperature, the HEA exhibited a YS of ~1660 MPa, more than twice that of pure tungsten. Although the YS decreased to ~650 MPa at 500 °C, it was more than 2.5 times higher than tungsten’s YS at the same temperature (~250 MPa), indicating excellent strength retention at high temperatures. The HEA exhibited greater plastic strain of ~6% below 100 °C compared to negligible plasticity for pure tungsten. The plastic strain of the HEA increased steadily with temperature, reaching over 20% at 500 °C. The higher plastic strain for the HEA may be attributed to the presence of ductile elements like Ti and V. The presence of Ti and Zr lowers the average valence electron concentration (VEC). For BCC alloys, a VEC below 4.5 has been associated with enhanced intrinsic ductility [35,36]. Additionally, Ti is known to improve grain boundary cohesion in W-based HEAs, suppressing inter-granular fracture and promoting trans-granular failure, which increases the alloy’s toughness [26]. In summary, the WTaTiVZr HEA showed a synergistic combination of ultra-high strength and relatively larger plastic strain compared to pure tungsten. W and Ta in the HEA contributed towards higher strength through lattice distortion and frictional resistance, while Ti, V, and Zr may have promoted better plasticity by dislocation core modification [7,9].

Figure 3b and Figure 3d show the optical images around indents for the WTaTiVZr alloy and pure tungsten, respectively. At lower temperatures (25 °C to 200 °C), small radial cracks were seen around the indent corners in WTaTiVZr (Figure 3b), indicating a brittle response. In contrast, no cracking was observed above 200 °C. The absence of cracking above 200 °C implies a transition towards a more ductile deformation mode. For pure tungsten (Figure 3d), cracks were visible up to 300 °C, whereas at 400 °C, the cracks disappeared, and a more uniform plastic flow was observed, suggesting a transition to a ductile deformation mode. The higher brittle-to-ductile transition temperature (BDTT) in tungsten may cause significant challenges for operational reliability in fusion reactor applications [3,37]. In this context, the as-cast WTaTiVZr offers a more reliable alternative due to its higher strength, relatively larger plastic strain, and lower BDTT compared to pure tungsten.

**Figure 4:**
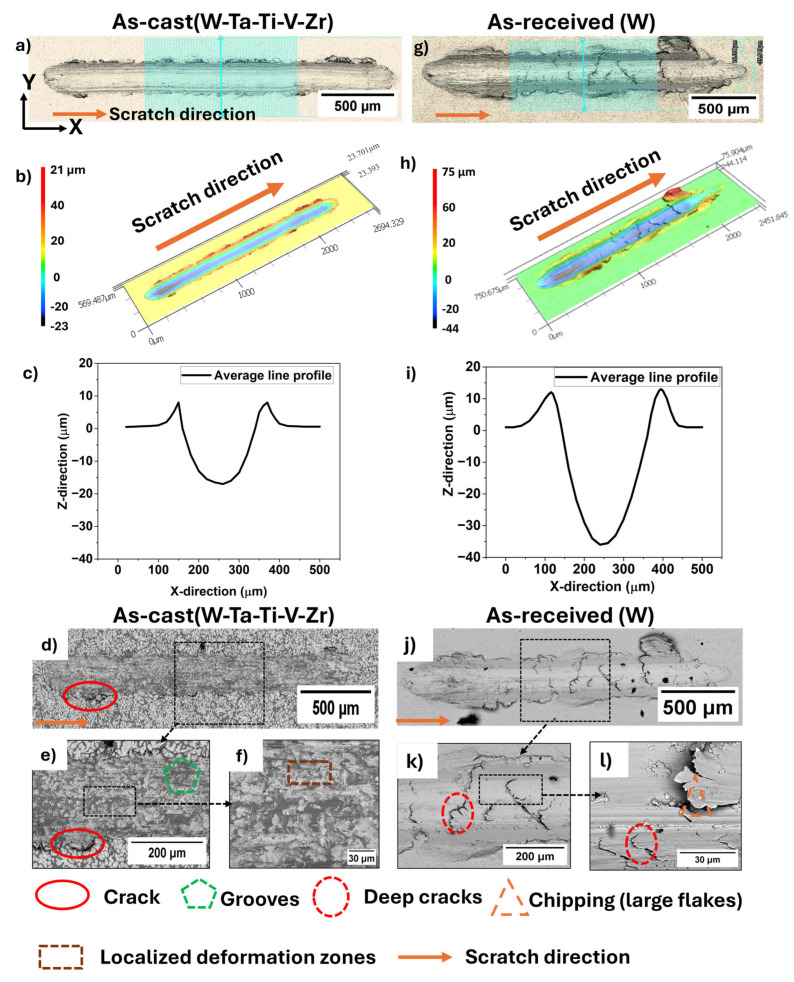
Post-scratch analysis of (**a**–**f**) as-cast WTaTiVZr alloy and (**g**–**l**) pure W. (**a**,**g**) Confocal laser microscope images with 100 line profiles (10 µm apart, in blue) used to extract groove profiles; (**b**,**h**) 3D profiles of scratch groove; (**c**,**i**) depth profiles of scratch along YZ plane. SEM images of the scratch grooves for (**d**–**f**) as-cast WTaTiVZr alloy and (**j**–**l**) pure W.

Figure 4 shows the scratch response of the as-cast WTaTiVZr HEA and commercially used pure tungsten. Figure 4a–c and Figure 4g–i show the scratch groove images, 3D profiles, and line scans of the as-cast HEA and pure tungsten, respectively. To capture a representative cross-section along the YZ plane, 100-line profiles were taken along the X-direction (highlighted by the blue lines in Figure 4a,g) and averaged across three scratches per sample. These surface profiles were analyzed to extract the groove perimeter (*P*) and the projected contact area (*A_C_*) to calculate the fracture toughness (*K_C_*) values that are listed in Table 1.

The as-cast WTaTiVZr alloy showed shallow, smooth, and more uniform scratch grooves with minimal cracking (Figure 4a–c). In contrast, the pure tungsten sample (Figure 4g–i) showed deeper grooves with multiple cracks, both parallel and perpendicular to the scratch direction. The irregular groove shape and asymmetric pile-up indicate brittle behavior and limited plasticity for tungsten. Figure 4d–f and Figure 4j–l show the SEM images of the scratch grooves for the as-cast WTaTiVZr alloy and pure tungsten, respectively. Pure tungsten showed deep and sharp cracks as well as visible chipping along the edge of the scratch groove, indicating brittle fracture. This brittle behavior is often observed in coarse-grained tungsten, where limited dislocation mobility at room temperature leads to cleavage fracture. In contrast, as-cast WTaTiVZr alloy showed a uniform groove with few cracks and significantly less surface damage (Figure 4d–f). The presence of multiple principal elements in the HEA promoted solid solution strengthening and lattice distortion, hindering dislocation motion and crack propagation [7,38]. Furthermore, the fragmented zones observed in Figure 4d–f may indicate localized plastic deformation and energy dissipation, contributing to the overall improved fracture resistance of the HEA. The fracture toughness (*K_C_*) increased by ~52%, from 25 MPa√m in pure tungsten to 38 MPa√m in the as-cast WTaTiVZr HEA.

## 4. Conclusions

The as-cast WTaTiVZr high-entropy alloy, composed of all low-activation elements, exhibited a dendritic microstructure with W–Ta-rich dendrites and Zr–Ti-V-rich inter-dendritic regions, both with body-centered cubic crystal structure. The alloy showed high compressive yield strength at room temperature of ~1.6 GPa and plastic strain ~6%. The fracture surfaces after compression revealed shear failure characterized by cleavage facets and river patterns, indicative of trans-granular fracture. The alloy exhibited superior high-temperature strength retention, maintaining a yield strength of approximately 650 MPa at 500 °C, which is ~160% higher than pure tungsten at the same temperature. Fracture toughness for the HEA was ~38 MPa√m compared to ~25 MPa√m for commercially used pure tungsten. This combination of thermal stability, high strength, and damage tolerance highlights its potential for structural applications in next-generation nuclear reactors.

## Figures and Tables

**Table 1:** Physical parameters from line profiles of scratch grooves for K_C_ determination.

Sample	Force (N)	Speed (mm/s)	Track Width (µm)	Area (µm^2^)	Perimeter (µm)	Depth (µm)	Fracture Toughness (*K_C_*) (MPa√m)
WTaTiVZr	100	0.01	198	2760	205	24	38 ± 1
Pure W	100	0.01	360	15,840	395	74	25 ± 1

## Data Availability

Data will be made available upon request.

## References

[1] Bolt H., Barabash V., Federici G., Linke J., Loarte A., Roth J., Sato K. (2002). Plasma facing and high heat flux materials Plasma facing and high heat flux materials-needs for ITER and beyond. J. Nucl. Mater..

[2] Nygren R.E., Rognlien T.D., Rensink M.E., Smolentsev S.S., Youssef M.Z., Sawan M.E., Merrill B.J., Eberle C., Fogarty P.J., Nelson B.E. (2004). A fusion reactor design with a liquid first wall and divertor. Fusion Eng. Des..

[3] Rieth M., Dudarev S.L., De Vicente S.G., Aktaa J., Ahlgren T., Antusch S., Armstrong D.E.J., Balden M., Baluc N., Barthe M.F. (2013). Recent progress in research on tungsten materials for nuclear fusion applications in Europe. J. Nucl. Mater..

[4] Gludovatz B., Wurster S., Weingartner T., Hoffmann A., Pippan R. (2011). Influence of impurities on the fracture behaviour of tungsten. Philos. Mag..

[5] Hu X., Koyanagi T., Fukuda M., Kumar N.A.P.K., Snead L.L., Wirth B.D., Katoh Y. (2016). Irradiation hardening of pure tungsten exposed to neutron irradiation. J. Nucl. Mater..

[6] Miracle D.B., Senkov O.N. (2017). A critical review of high entropy alloys and related concepts. Acta Mater..

[7] Senkov O.N., Miracle D.B., Chaput K.J., Couzinie J.P. (2018). Development and exploration of refractory high entropy alloys—A review. J. Mater. Res..

[8] Chen S., Qi C., Liu J., Zhang J., Wu Y. (2022). Recent Advances in W-Containing Refractory High-Entropy Alloys—An Overview. Entropy.

[9] Hatler C., Robin I., Kim H., Curtis N., Sun B., Aydogan E., Fensin S., Couet A., Martinez E., Thoma D.J. (2025). The path towards plasma facing components: A review of state-of-the-art in W-based refractory high-entropy alloys. Curr. Opin. Solid State Mater. Sci..

[10] Ayyagari A., Salloom R., Muskeri S., Mukherjee S. (2018). Low activation high entropy alloys for next generation nuclear applications. Materialia.

[11] Peterson D.T., Hull A.B., Loomis B.A. (1992). Hydrogen embrittlement considerations In niobium-base alloys for application in the ITER divertor. J. Nucl. Mater..

[12] Shi Y., Yang B., Liaw P.K. (2017). Corrosion-resistant high-entropy alloys: A review. Metals.

[13] Watanabe K., Hishinuma A., Hiraoka Y., Fujii T. (1998). Neutron irradiation embrittlement of polycrystalline and single crystalline molybdenum. J. Nucl. Mater..

[14] Zinkle S.J., Snead L.L. (2014). Designing radiation resistance in materials for fusion energy. Annu. Rev. Mater. Res..

[15] Sadeghilaridjani M., Muskeri S., Pole M., Mukherjee S. (2020). High-temperature nano-indentation creep of reduced activity high entropy alloys based on 4-5-6 elemental palette. Entropy.

[16] Patel K., Mahajan C., Muskeri S., Mukherjee S. (2023). Corrosion Behavior of Refractory High-Entropy Alloys in FLiNaK Molten Salts. Metals.

[17] Said G. (2006). Study on ASTM E399 and ASTM E1921 standards. Fatigue Fract. Eng. Mater. Struct..

[18] Wurmshuber M., Alfreider M., Wurster S., Burtscher M., Pippan R., Kiener D. (2023). Small-scale fracture mechanical investigations on grain boundary doped ultrafine-grained tungsten. Acta Mater..

[19] Jha S., Muskeri S., Alla S.S., Mukherjee S. (2023). Structural and stress state dependence of small-scale deformation in bulk metallic glass. J. Alloys Compd..

[20] Kong B.S., Shin J.H., Jang C., Kim H.C. (2020). Measurement of fracture toughness of pure tungsten using a small-sized compact tension specimen. Materials.

[21] Rupp D., Weygand S.M. (2010). Anisotropic fracture behaviour and brittle-to-ductile transition of polycrystalline tungsten. Philos. Mag..

[22] Akono A.T., Ulm F.J. (2011). Scratch test model for the determination of fracture toughness. Eng. Fract. Mech..

[23] Estrada K.A., Verma K.K., Sharma S., Argibay N., Vasudevan V.K., Dahotre N.B. (2025). Fracture toughness of additively manufactured tungsten-rhenium via surface scratch technique. Int. J. Refract. Metals Hard Mater..

[24] Clyne T.W., Campbell J.E., Burley M., Dean J. (2021). Profilometry-Based Inverse Finite Element Method Indentation Plastometry. Adv. Eng. Mater..

[25] Miller J.R., McKeown P.J., Qiu H., Clyne T.W. (2025). Profilometry-Based Indentation Plastometry Testing of Tungsten at High Temperature. Adv. Eng. Mater..

[26] Han Z.D., Luan H.W., Liu X., Chen N., Li X.Y., Shao Y., Yao K.F. (2018). Microstructures and mechanical properties of TixNbMoTaW refractory high-entropy alloys. Mater. Sci. Eng. A.

[27] Senkov O.N., Woodward C.F. (2011). Microstructure and properties of a refractory NbCrMo_0.5_Ta_0.5_TiZr alloy. Mater. Sci. Eng. A.

[28] George E.P., Curtin W.A., Tasan C.C. (2020). High entropy alloys: A focused review of mechanical properties and deformation mechanisms. Acta Mater..

[29] Zou Y., Ma H., Spolenak R. (2015). Ultrastrong ductile and stable high-entropy alloys at small scales. Nat. Commun..

[30] Moschetti M., Xu A., Schuh B., Hohenwarter A., Couzinié J.P., Kruzic J.J., Bhattacharyya D., Gludovatz B. (2020). On the Room-Temperature Mechanical Properties of an Ion-Irradiated TiZrNbHfTa Refractory High Entropy Alloy. Jom.

[31] Li H., Wurster S., Motz C., Romaner L., Ambrosch-Draxl C., Pippan R. (2012). Dislocation-core symmetry and slip planes in tungsten alloys: Ab initio calculations and microcantilever bending experiments. Acta Mater..

[32] Tarleton E., Roberts S.G. (2009). Dislocation dynamic modelling of the brittle-ductile transition in tungsten. Philos. Mag..

[33] Peter G., Joachim R., Alexander H., Hellmut F.F. (1998). Controlling Factors for the Brittle-to-Ductile Transition in Tungsten Single Crystals. Am. Assoc. Adv. Sci..

[34] Moschetti M., Burr P., Obbard E., Kruzic J.J., Hosemann P., Gludovatz B. (2022). Design considerations for high entropy alloys in advanced nuclear applications. J. Nucl. Mater..

[35] Sheikh S., Shafeie S., Hu Q., Ahlström J., Persson C., Veselý J., Zýka J., Klement U., Guo S. (2016). Alloy design for intrinsically ductile refractory high-entropy alloys. J. Appl. Phys..

[36] Zhang C., Wang H., Wang X., Tang Y.T., Yu Q., Zhu C., Xu M., Zhao S., Kou R., Wang X. (2023). Strong and ductile refractory high-entropy alloys with super formability. Acta Mater..

[37] Ren C., Fang Z.Z., Koopman M., Butler B., Paramore J., Middlemas S. (2018). Methods for improving ductility of tungsten—A review. Int. J. Refract. Metals Hard Mater..

[38] Pickering E.J., Carruthers A.W., Barron P.J., Middleburgh S.C., Armstrong D.E.J., Gandy A.S. (2021). High-entropy alloys for advanced nuclear applications. Entropy.

